# Application of Graph Convolutional Neural Networks Combined with Single-Model Decision-Making Fusion Neural Networks in Structural Damage Recognition

**DOI:** 10.3390/s23239327

**Published:** 2023-11-22

**Authors:** Xiaofei Li, Langxing Xu, Hainan Guo, Lu Yang

**Affiliations:** 1 College of Transportation Engineering, Dalian Maritime University, Dalian 116026, China; xlx@dlmu.edu.cn (L.X.); yl2501033920@dlmu.edu.cn (L.Y.); 2 Beijing Key Laboratory of Earthquake Engineering and Structure Retrofit, University of Technology Beijing, Beijing 100124, China; guohainan153@163.com

**Keywords:** graph convolutional neural networks, damage identification, decision-level data fusion, sensor spatial characteristics, model testing

## Abstract

In cases with a large number of sensors and complex spatial distribution, correctly learning the spatial characteristics of the sensors is vital for structural damage identification. Graph convolutional neural networks (GCNs), unlike other methods, have the ability to learn the spatial characteristics of the sensors, which is targeted at the above problems in structural damage identification. However, under the influence of environmental interference, sensor instability, and other factors, part of the vibration signal can easily change its fundamental characteristics, and there is a possibility of misjudging structural damage. Therefore, on the basis of building a high-performance graphical convolutional deep learning model, this paper considers the integration of data fusion technology in the model decision-making layer and proposes a single-model decision-making fusion neural network (S_DFNN) model. Through experiments involving the frame model and the self-designed cable-stayed bridge model, it is concluded that this method has a better performance of damage recognition for different structures, and the accuracy is improved based on a single model and has good damage recognition performance. The method has better damage identification performance in different structures, and the accuracy rate is improved based on the single model, which has a very good damage identification effect. It proves that the structural damage diagnosis method proposed in this paper with data fusion technology combined with deep learning has a strong generalization ability and has great potential in structural damage diagnosis.

## 1. Introduction

In the field of structural damage diagnosis, the spatial structure of sensors is crucial for understanding structural behavior and identifying damage. In large civil infrastructures, the large number of sensors and their complex spatial distribution make damage recognition difficult. Deep learning techniques have excellent capabilities in structural damage recognition, using deep network structures and large-scale training datasets which can automatically learn and extract multilevel and high-order features from the data, thus realizing accurate recognition and localization of structural damages [1,2,3,4,5,6]. However, methods such as 1D-CNN, RNN, etc. have some limitations in capturing the spatial structural relationships between sensors. Graph convolutional neural networks are a deep learning model for processing graph-structured data which can be utilized to aggregate the spatial information of sensors and their interconnectivity to learn more informative features from the data. By utilizing the spatial information encoded in the graph structure of the sensor network, GCNs can effectively enhance the vibration signals, facilitate the mapping between the vibration signals and the type of structural damage, and learn the spatial features of the sensors, which has the advantages of automatically extracting the feature information and high accuracy to cope with such problems.

Although GCN models have been widely applied and studied in many other industry fields, such as social networks, computer vision, and natural language processing, there are still relatively few studies on extracting the features of vibration signals using GCN models and using them for structural damage recognition. The application of the GCN model can help to solve the problem of unclear relationships between sensor networks existing in Structure Health Monitoring (SHM). The information transfer in the sensor network can be better modeled by the GCN model, thus improving the accuracy and efficiency of structural damage identification. Zhicheng Liu [7] proposed a health monitoring model for railway structures based on graph neural networks which further extracts the temporal information between individual sensors through one-dimensional convolution, processes the spatial structural information between the sensor network and the damage nodes, predicts the damage location, and uses recurrent neural networks for multi-damage node identification. The superiority of the model was verified on a simulation dataset based on a plate structure, but the dataset is small and a larger dataset is needed for future verification analysis to enhance the performance of the graph neural network to improve the model’s capability. Dang et al. [8] proposed a g-SDDL model that utilizes graph neural networks to capture the spatial correlation of sensor positions and extract vibration signal patterns through convolutional operations, and the feasibility of this method was verified through three case studies, where it consistently achieved a high damage detection accuracy of more than 90% in complex environments. This method is the first time that a GCN has been applied to feature extraction of structural vibration signals and structural damage type identification, but the application of this method in the field of structural damage identification is still in its infancy and requires further research and exploration.

In the identification stage, the new response data of the structure is collected, and the trained model is used to predict the type of damage. However, even very good network models cannot completely and accurately identify the damage occurring in the structure; all of them have certain defects, and some of the vibration signals are prone to be altered from their basic characteristics due to the influence of environmental disturbances, instability of the sensors, etc., which causes the possibility of misjudging the damage to the structure. Data fusion techniques can combine data from multiple sources and relevant information from related databases to achieve higher accuracy and more specific inferences than can be achieved by using a single source of information alone. Generally, the data fusion process is categorized into three levels: low, medium, and high, depending on the processing stage at which the fusion occurs. Among them, high-level fusion, also known as decision-level fusion, combines the results of multiple decisions to form a final decision result. In the field of SHM, many scholars have applied data fusion techniques to structural damage identification methods and achieved some research results. Grande et al. [9] proposed a linear system damage identification method by data fusion of modal strain energy from different information sources. Zhou et al. [10] used DS evidence theory to fuse the outputs of a posteriori probabilistic support vector machine for decision-level information fusion in building structural damage detection. Guo et al. [11] utilized multi-scale space theory and data fusion methods for multiple damage detection in beam structures and slab structures. The neural network has strong self-learning, self-organization, and self-adaptation capabilities, and can simulate complex nonlinear mapping relationships, which precisely meets the processing requirements of the fusion system and can realize decision-making fusion for structural damage identification.

In this paper, the decision-level data fusion technique is fully combined with the advantages of the GCN model in extracting vibration signal features by establishing different sensor network relationships, and a single-model Decision Fusion Neural Networks model is established to enhance the robustness of structural damage diagnosis methods. The main contributions of this paper are as follows:

1. A graph neural network is introduced to show how the positional relationships of the sensors are fed into the network structure, and it is verified that the GCN model has an advantage over the 1D-CNN model in terms of recognition accuracy due to its ability to mine the spatial structure of the sensors;
2. Based on building a high-performance deep learning model, this paper considers incorporating data fusion techniques at the model decision layer to further improve the accuracy and robustness of the model;
3. Based on the frame structure model and the self-designed cable-stayed bridge test model of two structures, experimental validation is carried out, which proves that the method has a certain generalization ability and can mine the spatial characteristics of the structure, correctly identify the structural damage, and has a certain improvement in the performance relative to a single GCN.

The rest of the paper is organized as follows: Section 2 describes the theoretical foundations, including graph neural networks and data fusion techniques, while detailing the specific architecture of the neural network model proposed in this paper; Section 3 describes the two architectures used for the experiments along with specific discussion of the results; and Section 4 gives the conclusions.

**Figure 1:**
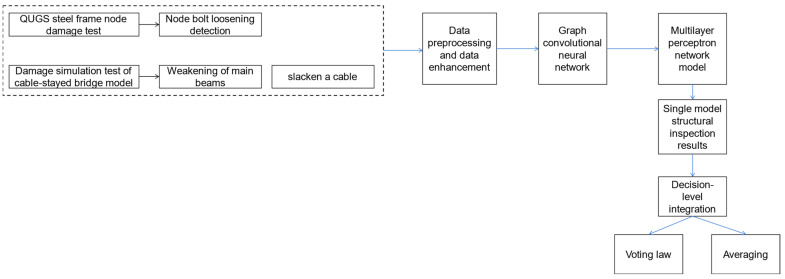
The overview of the methodology.

The organizational framework of the proposed approach is shown in Figure 1, which outlines the research methodology of this paper. We obtain damage signals from the framework model and cable-stayed bridge model, preprocess and enhance the signals, and then input them into the neural network for training. Based on the different damage problems, we choose the mean method or voting method for data fusion, which will be described in detail in the following subsections.

## 2. Materials and Methods

**Figure 2:**
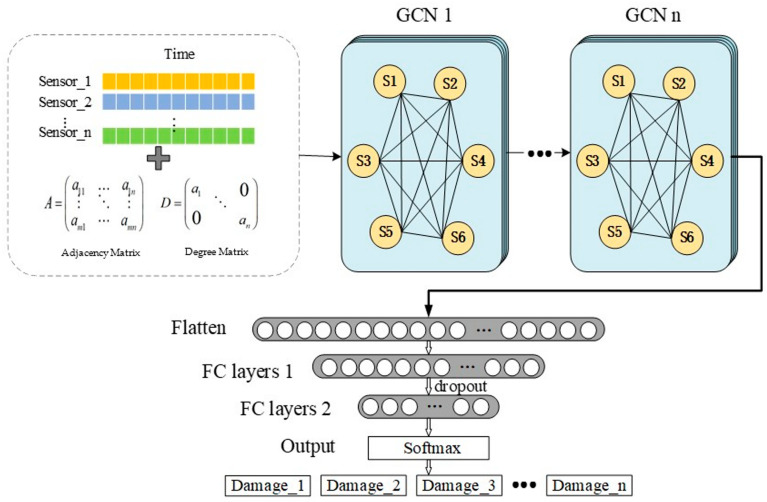
GCN model architecture for structural damage diagnosis.

This article feeds the spatial characteristics between sensors into a GCN. In the absence of edge connections between adjacent sensors, a sensor mapping network is first established based on the spatial characteristics of the sensors in the model. Then, the adjacency matrix and degree matrix of the sensor network are established to characterize the spatial structural relationship between sensor nodes. Then, the adjacency matrix and sensor array signal are simultaneously input into the network. Finally, the extracted vibration signal features are input into a multi-layer perceptron decision maker (MLP) for damage diagnosis decision-making. The model architecture consists of a graph convolutional neural network module and a multi-layer perceptron module. The former is mainly used to extract feature data that can better represent damage from structural acceleration data, while the latter is mainly used to establish a mapping relationship between feature data and damage types, achieving a structural damage diagnosis function. Figure 2 shows the basic architecture of the proposed deep learning model. The theoretical and functional aspects of each layer will be described in detail in the following sections.

### 2.1. GCN Module

The image convolution feature extraction module is constructed by overlaying multiple image convolution layers, as shown in Figure 2.

The GCN extracts the feature representation of a node by learning the relationships between the nodes to represent and classify the entire graph. The core idea of GCNs is to aggregate the features of the node’s neighboring nodes, and then linearly transform and nonlinearly activate the aggregated features to obtain a new node representation.

**Figure 3:**
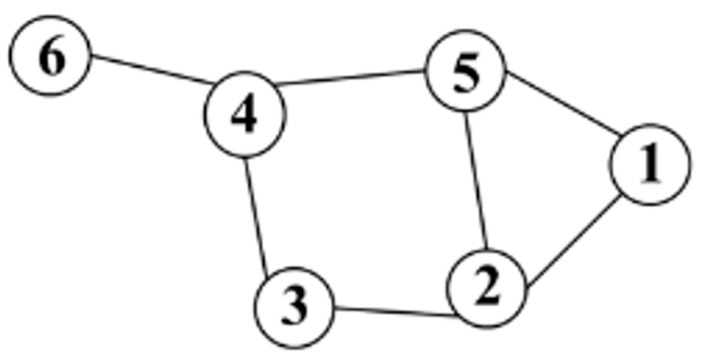
Structure of the mapping network.

Given an undirected network *G* = (*V*, *E*), where *V* contains all N nodes, i.e., *vi* ∈ *V*, *E* denotes the edges between the nodes, (*vi*, *vj*) ∈ *E*, and the elements of the adjacency matrix *A* ∈ *R^N^*^×*N*^ can be either binary encoded or weighted real numbers (cf. Equation (1)), the adjacency matrix can be solved to find the degree matrix D, which is a diagonal array where the diagonal elements of the degree matrix can be computed by Equation (2). As shown in Figure 3, there are 6 nodes in total. According to the network structure constructed adjacency matrix A, the degree matrix D can be calculated from Equation (2).

(1) $$A=\left(\begin{array}{ccc} a_{11} & \ldots & a_{1n}\\ \vdots & \ddots & \vdots \\ a_{m1} & \cdots & a_{mn} \end{array}\right)$$

(2) $$D_{ii}=\sum_{j=1}^{N}A_{ij}$$

$$A=\left[\begin{array}{cccccc} 0 & 1 & 0 & 0 & 1 & 0\\ 1 & 0 & 1 & 0 & 1 & 0\\ 0 & 1 & 0 & 1 & 0 & 0\\ 0 & 0 & 1 & 0 & 1 & 1\\ 1 & 1 & 0 & 1 & 0 & 0\\ 0 & 0 & 0 & 1 & 0 & 0 \end{array}\right]$$

$$D=\left[\begin{array}{cccccc} 2 & 0 & 0 & 0 & 0 & 0\\ 0 & 3 & 0 & 0 & 0 & 0\\ 0 & 0 & 2 & 0 & 0 & 0\\ 0 & 0 & 0 & 3 & 0 & 0\\ 0 & 0 & 0 & 0 & 3 & 0\\ 0 & 0 & 0 & 0 & 0 & 1 \end{array}\right]$$

As Ref. [12] described, in the linear formulation of the GCN, by replacing the convolution kernel in the spectral domain with Chebyshev polynomials, and then considering only Chebyshev polynomials of the first order, and further approximating *λ*max ≈ 2, the Laplacian matrix of the graph structure can be expressed as:

(3) L=D−A

Then, the symmetric normalized Laplace operator is expressed as:

(4) $$L_{s}=I_{N}-D^{-\frac{1}{2}}AD^{-\frac{1}{2}}$$

If this operator is used in a deep neural network model, repeated application of the operator leads to numerical instability (divergence) and gradient explosion or vanishing. To solve this problem, a re-normalization trick is introduced:

(5) $$L_{s}=I_{N}-D^{-\frac{1}{2}}AD^{-\frac{1}{2}}\to {\tilde{D}}^{-\frac{1}{2}}A{\tilde{D}}^{-\frac{1}{2}}$$

As shown in the above formulas, the processing of graph signals, Laplace matrix, and Fourier transform are introduced into the GCN to obtain mathematical expressions for each layer of the GCN:

(6) $$H^{\left(l+1\right)}=\sigma \left({\tilde{D}}^{-\frac{1}{2}}\tilde{A}{\tilde{D}}^{-\frac{1}{2}}H^{\left(l\right)}W^{\left(l\right)}\right)$$

where *H^l^* denotes the lth layer in the network, *σ* is the activation function of the represented nonlinearity, and *W* denotes the weight matrix of the current hidden layer. $\tilde{D}$ and $\tilde{A}$ are the degree matrix and adjacency matrix of the graph, respectively.

By researching relevant literature and data, and conducting corresponding experimental tests, it was found through comparison that a three-layer GCN feature extraction module is more suitable for the structural damage diagnosis research in this article. Therefore, this article adopts a three-layer GCN model and adds BN and Dropout layers to improve the model’s generalization ability and avoid overfitting. The specific selection method of GCN model parameters is described in the corresponding summary of each model.

### 2.2. MLP Module

The MLP decision module is mainly used to establish the mapping relationship between feature data and damage type to realize the function of damage decision. In this paper, two fully connected layers are selected to form this module. The number of neurons in the first layer (FC layer 1) is set to 256, and the activation function of this layer adopts the ReLU function. The second layer (FC layer 2) is the output layer, and the number of neurons is equal to the dimension of the damage labels. When classifying the type of damage, the output layer adopts the Softmax function, and the loss function corresponding to it adopts the categorical cross-entropy function, the mathematical expression of which is shown in Equation (7). The sample labels are encoded using one-hot coding; for example, the dataset has a total of n types, and when the sample belongs to type 1, the label encoding contains n elements, with the first element valued at 1, and the rest of the elements valued at 0, i.e., the label encoding is (1, 0, 0, 0, …, 0). In the damage degree regression analysis, the linear function is used as the output layer, and the corresponding loss function selected is the Mean Square Error (MSE) function, whose formula is shown in Equation (8). At this time, the label coding needs to consider the structural unit location and the degree of unit change. For example, when a certain structure consists of n positions that need to be detected, the 1st position change degree is 0.5 while the rest of the positions do not change. The sample label can be encoded as (0.5, 0, 0, 0, 0, …, 0), and the label form contains the structural damage location and the degree of damage. The method can learn the feature representations of the different types of damage, and these will be fused with the extracted features from the deep learning feature extraction module. Finally, the damage location will be output.

(7) $$loss=-\sum_{i=1}^{n}y_{i}•\log\bar{y_{i}}$$

(8) $$MSE=\frac{\sum_{i=1}^{n}\left(\bar{y_{i}}-y_{i}\right)}{n}$$

where y denotes the true label vector, $\bar{y}$ denotes the model predicted label vector, and *n* is the dimension of the label vector.

### 2.3. Decision Level Data Fusion

In the identification stage, the new response data of the structure is collected, and the trained model is used to predict the type of damage. However, even very excellent network models cannot completely and accurately identify the damage that occurs in a structure. All of them will have certain defects, and some of the vibration signals are prone to alterations that change their basic characteristics under the influence of environmental disturbances, sensor instability, and other factors, which results in the possibility of misjudging the damage to the structure [13,14,15,16,17,18]. Therefore, based on building a high-performance deep learning model, this paper considers the integration of data fusion technology in the model decision-making layer and proposes a single model decision-making fusion neural network model.

The proposed single-model decision-level data fusion refers to the use of certain damage identification methods to process multiple sets of sensor data from the same shorter time period for damage identification. The resulting damage identification results are fused to distill the overall comprehensive damage identification information, and ultimately to make the highest-level decision, which can improve the prediction accuracy and robustness of a single model. In order to avoid problems such as local anomalies, missing data, and noise in the monitoring data, we collect acceleration signals for relatively long time periods during the detection phase and use data slicing methods to generate multiple simultaneous detection samples. These samples are used as inputs to the deep learning model to obtain multiple decision results. Combining multiple decision results, we can derive the structural state of the time period.

In the process of decision-level fusion of deep learning models, one of the key aspects is to choose an optimal binding module. For the multi-label classification problem, the voting method is the most commonly used binding method, which mainly includes the absolute majority voting method, the relative majority voting method, the weighted voting method, and the soft voting method. Suppose there are *T* classifier models (*h*_1_, *h*_2_, …, *h_T_*) and l category labels (*c*_1_, *c*_2_, …, *c_l_*), whose mathematical expressions are:

(9) $$H(x)=C_{argmax}(\sum_{i=1}^{T}h_{i}^{1}(x),\cdots ,\sum_{i=1}^{T}h_{i}^{l}(x))$$

where *H* (*x*) is the final output of the synthesized decision result and $C_{argmax}$ is the return maximum index function.

When the degree of structural damage is considered, the damage detection problem belongs to the regression problem. When the decision layer fusion chooses the combination method of mean values, assuming that there are *T* regression prediction models, each model outputs the decision as (*h*_1_, *h*_2_, …, *h_T_*) and the label of the degree of change of the n positions (*d*_1_, *d*_2_, …, *d_n_*). The mathematical expression of this is:

(10) $$H(x)=\frac{1}{T}(\sum_{i=1}^{T}h_{i}^{1}(x),\cdots ,\sum_{i=1}^{T}h_{i}^{n}(x))$$

**Figure 4:**
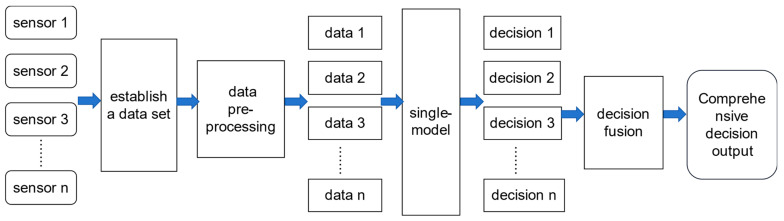
Single model combined with data fusion technique identification method.

In this paper, the data fusion technique is applied as Figure 4, the data within a fixed time period *T* s are continuously collected by a dynamic signal acquisition system for identifying the type of structural damage, and the sampling frequency is set to *f* Hz. A single *T* s data sample is augmented to n data samples by a sliding window slicing method. These samples are used as inputs for n model recognizers. The outputs of n damage labeling codes are outputted, and these recognition results are defaulted to the structural state assessment results in the same time period, and the transient time period structural state changes. The next step is to use the data fusion calculation method to synthesize the information of the n damage label codes to give the final structural damage diagnosis results. We consider two data fusion calculation methods, which are the mean value method and the voting method. When only considering identifying the location of structural damage, it belongs to the multi-task classification identification, and the use of the voting method can efficiently and stably compute the comprehensive results. When it is necessary to identify the location of structural damage and the degree of damage, addressing this requires solving a regression problem. The mean value method is more suitable for the output layer result data fusion.

## 3. Introduction to the Test

To investigate the effectiveness of the deep learning-based structural damage detection method proposed in this paper in recognizing different structural systems, a frame structure and a cable-stayed bridge structure are used, while different structural damage conditions are set up for the cable-stayed bridge structure and vibration signals are collected to build up the dataset. The following subsections describe the two test models and test schemes in detail.

### 3.1. Verification of Nodal Damage Identification in Steel Frame Structures

#### 3.1.1. Qatar University Grandstand Simulator Basics

**Figure 5:**
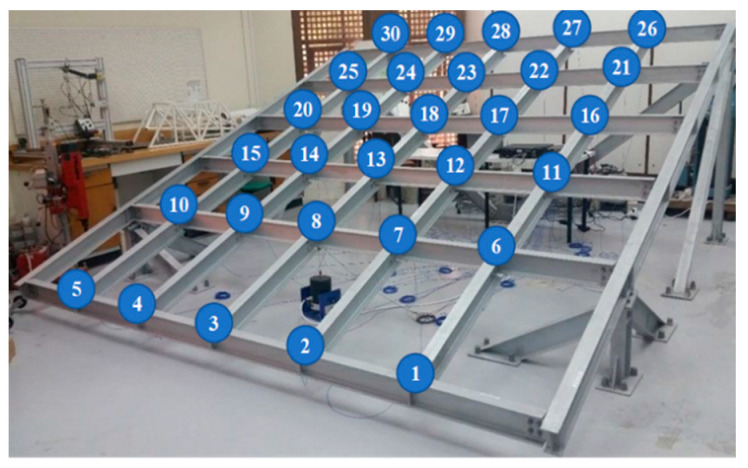
The Qatar University Grandstand Simulator. (The number is the number of 30 sensors).

In order to verify the effectiveness and generality of the proposed method for damage detection in different structures, a large frame test structure designed and constructed at the Structures Laboratory of Qatar University, which mainly consists of 8 main beams and 25 bracing units connected by 42 bolted nodes, was chosen as the object of study. The mechanism simulates the frame structure damage by releasing the node bolts. The experiment contains 31 test conditions, one of which is the healthy condition, and 30 damage conditions. The 30 damage conditions correspond to 30 instances of single node damage, and the specific damage locations are as follows. The specific damage locations are shown in Figure 5. Two tests were conducted for each test condition, with the random vibration occurring in the shaker as the excitation. In order to collect the structural acceleration data, 30 accelerometers were installed at the 30 nodes of the frame structure where the damage locations were set (numbered positions in Figure 5). The two experiments were recorded for a length of 256 s, with a sampling frequency of 1024 Hz. Each sensor recorded 262,144 samples, so two datasets, A and B, were established, each containing 8,126,464 samples.

#### 3.1.2. Model Parameters and Recognition Effectiveness

For the GCN model, the spatial features between different sensors can be mined and used to process the acceleration signal matrix samples collected by 30 sensor arrays, so all the sensor data of measurement points from No. 1 to No. 30 are used as the detection samples.

Using the GCN model for frame structure node damage detection, firstly, the neighbor matrix of the frame structure is established. By default, there is an association between the two sensors with the closest distance, and a total of 30 acceleration sensors are arranged in the structure. The neighbor matrix with 30 × 30 dimensions is established, and the degree matrix is calculated through Equation (3). Finally, the Laplacian operator for the structure of the sensor network is computed through Equation (7), and it is involved in the GCN model training. Then, the GCN model is discussed for the three-layer structure stacking.

The GCN model requires fewer hyperparameters, and the most important parameters are the output feature size parameter and the batch size parameter in each layer of the structure. In addition, each layer of the structure adopts the ReLU activation function. During the training process, the optimizer uses the Adam function.

**Figure 6:**
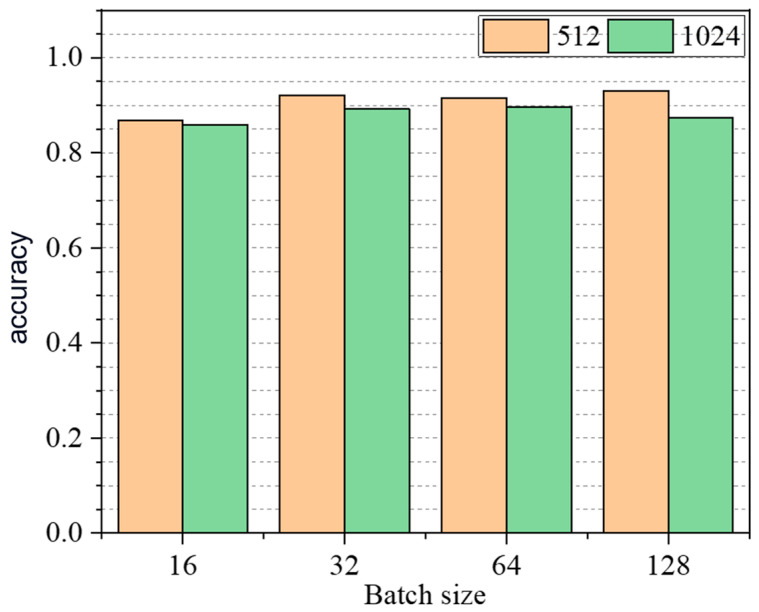
Recognition results of joint damage by GCN model with different parameters.

In order to analyze the impact of the two parameters of layer output feature size and batch size in the layer structure on the recognition accuracy of the GCN model, the output feature size values are taken as 512, 1024, and 2048, and the batch size values are taken as 16, 32, 64, and 128. Finally, 500 epochs of training are carried out using dataset A. When the output feature size is selected as 2048, the model gradient cannot be decreased, so it is no longer used. A total of eight GCN models were trained, and validated by the data in dataset B. The recognition accuracy of the eight models is shown as follows in Figure 6. When the output feature size value is 512, the batch size value is 128 and the recognition accuracy of the GCN model is the highest; at this time, the accuracy rate is 93.03%. The finalized hyperparameters of the GCN model for frame structures are shown as follows in Table 1.

To demonstrate the superiority of this method, the recognition accuracy of this method is compared with other common neural networks. One CNN model that can effectively extract features from sequence data is 1D-CNN. Unlike traditional RNNs, 1D-CNN can process the entire sequence in parallel, resulting in faster computation speed and fewer parameters, making it a common and efficient neural network model. The use of LSTM for training and identification has achieved certain results in structural damage identification research in fields such as architecture, bridges, and offshore jackets. [19] Comparing the experimental results of this method with the two models mentioned above based on the common dataset, it can be found that the GCN model is more suitable for analyzing complex sensor structures, and its recognition performance is better than the 1D-CNN model and LSTM model. The specific identification accuracy of node damage is shown in Table 2.

#### 3.1.3. Noise Resistance

In practical applications, environmental noise will inevitably affect the quality of monitored vibration signals. In different environments, the form of noise also varies. To test the ability of the proposed method to resist noise, a method of reducing the signal-to-noise ratio (SNR) of the signal is used to simulate the impact of noise on the signal. The signal-to-noise ratio calculation formula is shown in Equation (11), and different noise levels are added to the acceleration response of the test dataset (see Equation (12)). We reduce the signal-to-noise ratio of the validation set signal, verify the recognition accuracy of the proposed method through the denoised validation set, and then analyze the anti-noise ability of the proposed method.

(11) SNR=10log10(PsignalPnoise)

(12) $$\hat{S_{n}}=S_{n}+\xi$$

(13) $$\xi ∼N\left(0,\sigma^{2}\right)$$

Among these, Sn is the signal test set without noise addition and $\hat{S_{n}}$ is the signal test set after adding noise, while ξ is a random variable that follows a Gaussian distribution, with zero mean and $\sigma^{2}$ variance. In this work, the values of noise σ to be added for signals with different signal-to-noise ratios were calculated using Equations (11)–(13). Seven signal-to-noise ratios of 80 dB, 60 dB, 40 dB, 20 dB, 15 dB, 10 dB, and 5 dB were selected to process and identify the validation sets of frame structure cases.

**Figure 7:**
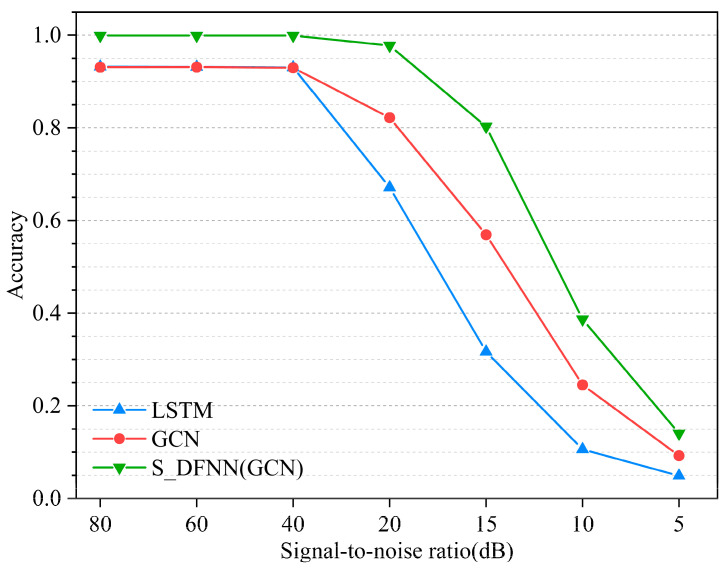
The recognition accuracy of some models for frame structure node damage at different SNRs.

In the case study of the frame structure under different signal-to-noise ratio conditions, the recognition results of each model for the frame structure test dataset are shown in Table 3. The recognition accuracy of some models for frame structure node damage is shown at different SNRs. As Figure 7 shows, when the signal-to-noise ratio is greater than 40 dB, the model maintains good recognition accuracy and has stable detection ability. In the range of signal-to-noise ratio from 40 dB to 20 dB, the recognition accuracy of each model began to decline in varying degrees. Among them, the S_DFNN (GCN) model has the least decline, and the LSTM model has the largest decline of 25.9%. When the signal-to-noise ratio of the verification set was lower than 20 dB, the recognition accuracy of the single model was less than 70%, which did not meet the actual demand. At a signal-to-noise ratio of 15 dB, only the S_DFNN (GCN) model still maintains good recognition performance, and the established data fusion neural network model not only improves the recognition accuracy of the model, but also improves its stability and noise resistance in damage detection.

### 3.2. Introduction to the Laboratory Cable-Stayed Bridge Test Model

In the pre-preparation stage of the test, the basic parameters of the test need to be determined. These parameters mainly include the overall arrangement parameters and specific structural parameters of the cable-stayed bridge model, the arrangement of measurement points in the model structure of the cable-stayed bridge, the simulation method of the excitation load, and the criteria for the baseline state (i.e., the state of health) of the cable-stayed bridge model. The following subsections will introduce the cable-stayed bridge test model and test program in detail.

**Figure 8:**
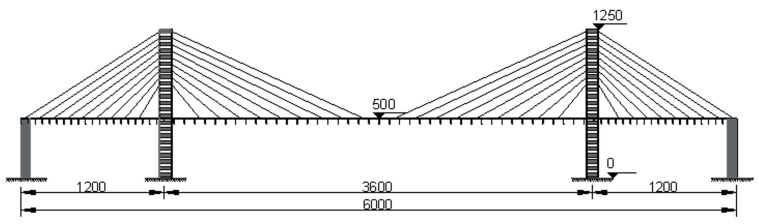
Parameters of the overall arrangement of the cable-stayed bridge model (unit: mm).

Under laboratory conditions requiring a maximum total length of 6 m, we established a model of a traditional double-tower three-span cable-stayed bridge. The main span of the cable-stayed bridge physical test model is 3600 mm with side spans of 1200 mm, resulting in a total bridge length of 6000 mm. The cable-stayed bridge model’s overall layout parameters are depicted in Figure 8. The overall arrangement parameters of the cable-stayed bridge model are shown in Figure 8.

**Figure 9:**
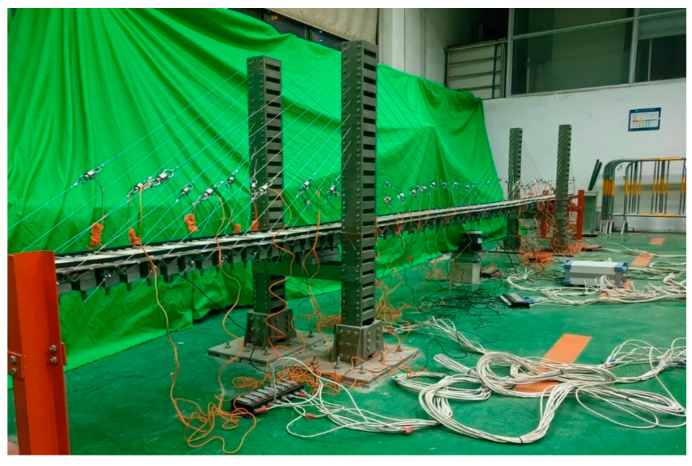
Test scene of cable-stayed bridge.

The shaker is used as the excitation load. The shaker can be controlled by the signal generator to simulate random waves through the dynamic signal acquisition system to collect the acceleration signal of the main beam of the cable-stayed bridge at 14 measurement points. The sampling frequency is set to 1000 Hz, and each condition is repeated for six tests. Each test process lasts for more than 100 s, and then 100 s of signal data are intercepted. Ultimately, each kind of condition used to obtain 600 s of acceleration signal data for the cable-stayed bridge test scene is shown in Figure 9. The test scene of the cable-stayed bridge is shown in Figure 9.

**Figure 10:**
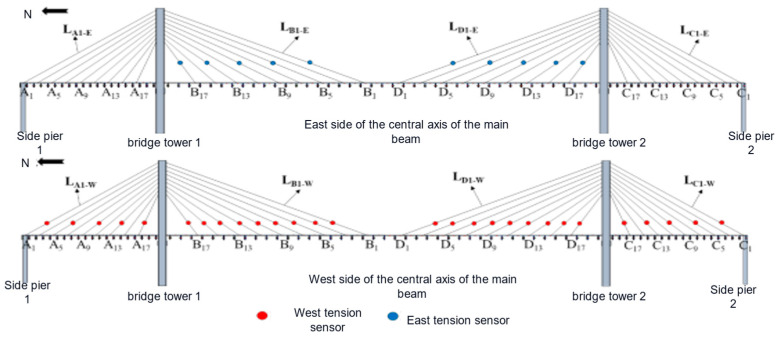
Layout of measuring points for tension sensors.

**Figure 11:**
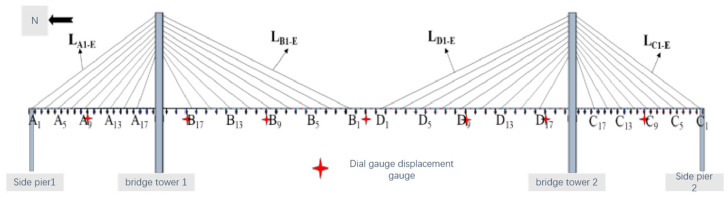
Micrometer point layout.

**Figure 12:**
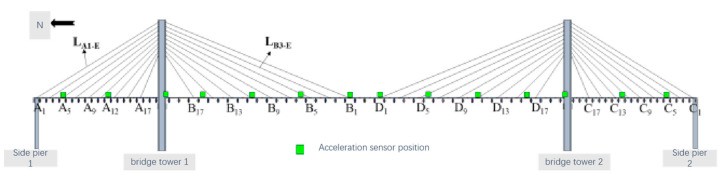
Acceleration sensor measurement point layout.

(1) Tension sensor measurement point arrangement: Due to the limited number of tension sensors, the demand for arranging tension sensors for each cable-stayed cable is not met. Therefore, through analysis, in order to achieve the purpose of regulating the rope force and controlling the overall state of the cable-stayed bridge, all the tension sensors are embedded in all the cable-stayed cables of the two sectors on the west side, arranged in sparse intervals. In addition, tension sensors are arranged on the east side of the cable-stayed cables to check the numerical values of the tension sensors on the west side with those on the east side. Numerical calibration, to a great extent, is used to monitor the overall state of the bridge rope force. Figure 10 shows the arrangement scheme of tension sensors on the sector tension cables on both sides of the center axis of the main beam, where a total of 38 tension sensors are arranged.
(2) Arrangement of micrometer measurement points: The arrangement of the micrometer is mainly used to monitor the deflection displacement of the key cross-section of the main girder. According to the parameters of the overall arrangement of the bridge model and finite element calculation and analysis, the selection is shown in Figure 11. At the same time, the displacement control of the key cross-section of the micrometer is coordinated with the horizontal filament positioning method, which is used for the zero displacement control of the main girder when the cable-stayed bridge is in a reasonable bridge condition.
(3) Acceleration sensor measurement point arrangement: When arranging the acceleration sensor, it is necessary to consider the sensitivity of the measurement point location for the structural modal changes. Combined with other structural analysis experience, the sensors will try to be arranged in the peak vibration position. The establishment of the cable-stayed bridge model of the finite element model involves modal analysis and the extraction of the model of the first ten orders of vertical vibration pattern. The sensors are placed at the peak vibration position. In the main girder unit we select the measurement point to set up a total of 14 acceleration sensors, as shown in Figure 12.

### 3.3. Design of Main Beam Damage Conditions

**Figure 13:**
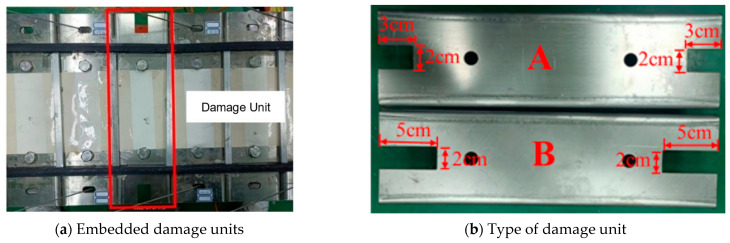
Main beam replacement damage unit.

**Figure 14:**
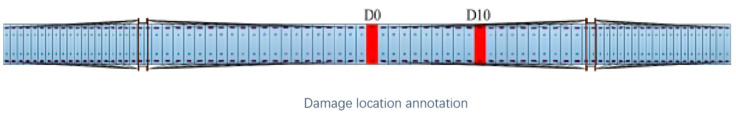
Location of damaged units of main beams.

The main girder of a cable-stayed bridge test model designed in the laboratory consists of 82 girder units, which are connected by bolts to realize the embedding of damaged girder units into the main girder structure, thus simulating the different damage conditions of the main girder. Through the design of cross-section cutting of the intact beam unit to simulate a damaged unit, this paper mainly designs two kinds of damage units: one with 6 cm of cutting damage, and another with 10 cm of cutting damage. The damage is distributed on the two sides of the main beam unit, as shown in Figure 13b which shows the type A damage unit and type B damage unit. These two kinds of damage units can be installed in different positions of the main beam to simulate a variety of main beam damage conditions. In this test, we mainly study the structural damage of the main span of the cable-stayed bridge model. We selected two positions of unit D0 and unit D10 for the replacement of the damage unit, as shown in Figure 14, and designed three kinds of damage conditions, which are described in the following table. The specific damage conditions are described in Table 4.

#### Analysis and Discussion of Main Beam Damage Identification Results

**Figure 15:**
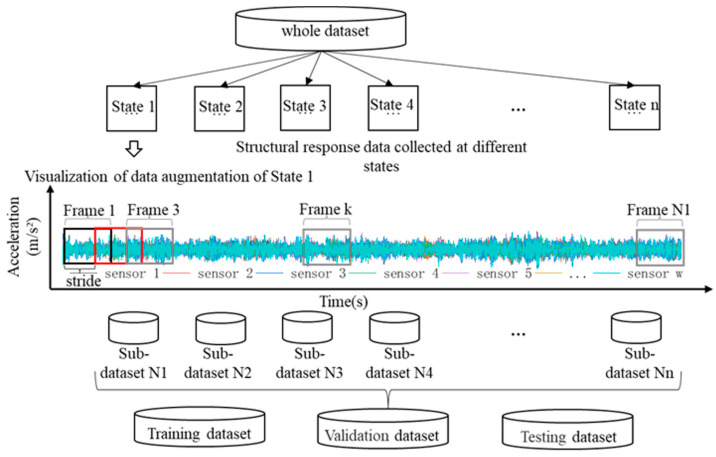
Data set preprocessing methods.

Dataset I, established by the cable-stayed bridge model test, is used to verify the accuracy of the model proposed in the previous section and its parameter configurations, and the data in dataset I are divided by data preprocessing methods. Normally, the structural response data under *n* states are monitored or collected by a signal acquisition system, all of which are multidimensional time series with a length of *L s* and a sampling frequency set to *f* Hz, whereas a certain batch of independent data samples of the same size is needed to meet the requirements of the network training in the process of deep learning network training, rather than a complete long-period time series. Therefore, it is necessary to use the sliding window method for data enhancement. The sliding window decomposition is a common method of signal data decomposition, and its division of the data set is shown in Figure 15; that is, the completed data are divided into *N* identical sub-data samples through a window of size *h × w* (window length, i.e., time dimension, is *h*, and window width, i.e., sensor dimension, is *w*) with a sliding time step of *s*. When the number of data is limited, the effect of data enhancement can be realized in the form of overlap of the two neighboring windows, and the total sample size of the dataset, *N*, can be computed by Equation (14):

(14) N=(1s×(Lf−h))+1

In order to accelerate the network training convergence speed and generalization ability, the data normalization process needs to be performed before data decomposition, as shown in Equation (6):

(15) $$\hat{L}=\frac{L_{0}-\bar{L_{0}}}{\sigma (L_{0})}$$

where $L_{0}$ is the original dataset, $\bar{L_{0}}$ and $\sigma \;(L_{0})$ are the mean and standard deviation of the original data respectively, and $\hat{L}$ is the normalized dataset.

Before training the deep learning model, the complete dataset collected from the experiment needs to be preprocessed. Firstly, the data are normalized on the same scale by the Equation (6) method, and the normalized data are augmented by the sliding window decomposition technique for data enhancement. A total of 600 s of data are collected for each scenario, of which two-thirds of the data samples (400 s) are used for model optimization and training, and the remaining one-third of the data samples (200 s) are used for cross-validation of the model performance. During the iterative training of the model, two-thirds of the dataset used for training is divided into a training set and a test set in the ratio of 4:1. The dataset inputs are often batch-processed, with each training session randomizing the order of the mixing batches according to the law of uniform distribution to ensure that all signals have an equal chance of being selected for training and testing.

For the GCN feature extraction module, the sliding window size is 500 × 14, and the sliding window step is 500. Each kind of working condition data in the dataset is divided into 1200 samples, of which 800 samples are used for model training and 400 samples are used for model validation. The ratio of the training set to the test set is 0.8:0.2. The eight models proposed in this paper are trained by the divided dataset and validated by the validation set data. The parameter configuration of the GCN model used to identify structural changes in cable-stayed bridges is shown in Table 5.

**Figure 16:**
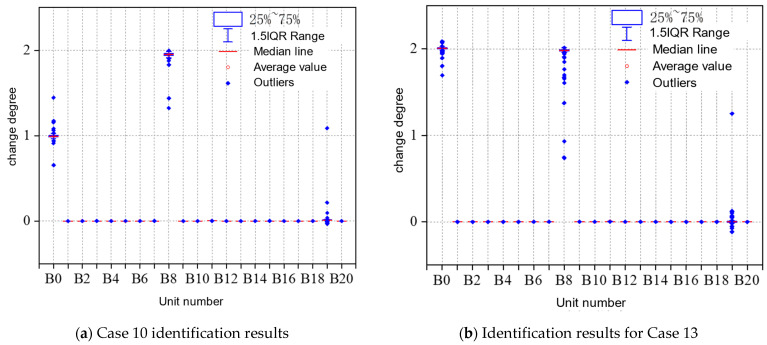
Regression prediction results of GCN model for main beam variation.

**Figure 17:**
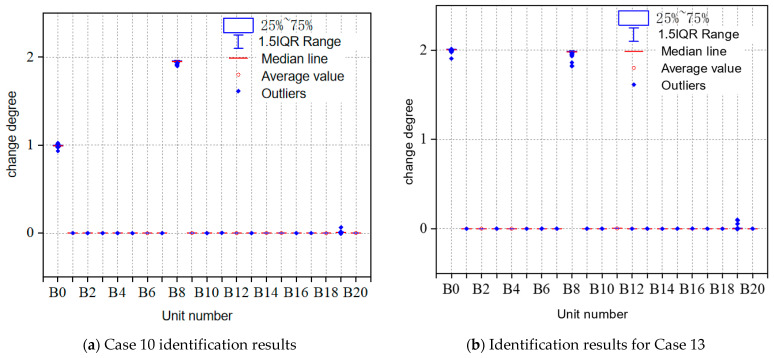
Regression prediction results of GCN-based S_DFNN model for main beam variation.

All the models for different main beam unit damage recognition accuracy are shown in Table 6. As shown in Figure 16 and Figure 17, from the overall recognition accuracy, the GCN model has a lower recognition accuracy, and the S_DFNN (GCN) model has an overall better recognition accuracy than the three single models, which can completely and accurately recognize the damage of each working condition.

### 3.4. Design of Damage Conditions of the Cable

**Figure 18:**
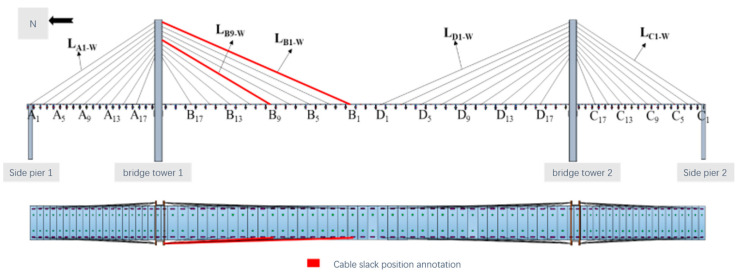
Tie slack position.

Stayed cables are one of the main load-bearing structures of cable-stayed bridge structures, and under long-term operation state, stayed cables are prone to structural weakening, relaxation, and other damage under long-term loading and environmental effects. In this section, the state of the tension cable is adjusted by the hollow bolt anchor cable structure, which is selected as shown in Figure 18. The slackening position of tension cables LB1_W and LB9_W as damage objects was designed as shown in Table 7. The injury conditions of inclined cables, including three typical damage conditions, were designed as shown in Table 7. Using the test method described in Section 3.1 above, a total of 600 s of acceleration signal data were obtained for each working condition, and a data set was established for the identification of structural relaxation damage of the cable structure, named Data Set III.

#### Analysis and Discussion of Cable Damage Identification Results

After model parameter optimization and several tests using the above hyperparameters, data set III was divided into a training set, test set, and validation set by the data preprocessing method adopted in the previous section. The GCN and S_DFNN (GCN) models are trained through the training set and test set, and the trained and optimized models are used to identify the three working conditions of the damage of the diagonal cable. The identification results are shown in Table 8. As shown in Table 8, the recognition accuracy of the three models does not exceed 90%, but after combining the data fusion technology, the models show excellent recognition ability, and the S_DFNN model based on a single model has a higher accuracy rate. From a comprehensive point of view, data fusion technology is effective in improving the recognition accuracy of the deep learning model for diagonal cable damage.

## 4. Conclusions and Outlook

The GCN model takes into account the correlation between different dimensions of data in multi-dimensional time series. It is suitable for mining the features of vibration signals collected by sensor array network from the dimension of spatial structure, and is able to address the complexity of spatial characteristics of sensors. This paper proposes to combine the GCN model with the S_DFNN technology to improve its stability and recognition accuracy, and further solve the damage miscalculation due to environmental factors. The specific conclusions are as follows:

- We demonstrated that the GCN model has certain advantages through the framework model case, and at the same time, can further improve the recognition accuracy when combined with the decision-level data fusion technology. The GCN reaches 93.03% accuracy, which exceeds the 1D-CNN model with data compression, and after combining it with the S_DFNN model, the model’s recognition accuracy reaches 99.57%, which is a substantial improvement;
- The main girder damage identification and stayed cable relaxation damage identification for the cable-stayed bridge model revealed that the GCN model is more sensitive to the structural change of location, and that the spatial characteristics between the sensor arrays can be mined, but there is room for improvement in identification performance and accuracy. After integrating the S_DFNN model, the accuracy of main girder damage recognition for cable-stayed bridges was improved by 11.44%;
- For the damage identification of the diagonal cable, the combination of decision-level data fusion technology and a deep learning model approach optimizes the identification accuracy of a single model to a certain extent and improves the identification accuracy of the structure by 21.81% to 99.69%, which can achieve the goal of accurately determining the damage to the diagonal cable.

In summary, combining the GCN model with the S_DFNN model can improve the recognition accuracy of the GCN model, and it has a good damage recognition effect on the application of different models with good generalization ability and robustness, which is an advanced and advantageous damage recognition method.

The application of the GCN model in structural damage recognition research is still relatively small. During the research of this paper, it was found that its application in simple sensor network structure scenarios is not ideal, but it shows an excellent ability for the damage of nodes of the frame structure with complex sensor network structure. Therefore, the study of a reasonable sensor network arrangement will improve the recognition accuracy of the GCN model, and at the same time, the study of a more accurate neighbor matrix establishment method is also very important. For large-span bridge structures such as cable-stayed bridges and suspension bridges, there are close interactions between different tension cables, and it is a worthwhile direction to explore and study the application of the GCN model to the damage of cable-stayed structures by using the cable-stay force as an evaluation index.

## Figures and Tables

**Table 1:** Parameter configuration of GCN model for damage identification of frame structure.

Parameter	Specific Values
First GCN layer	512
Second GCN layer	512
Third GCN layer	512
Dropout value	0.3
Training Optimizer	Adam

**Table 2:** Comparison of QUGS structural damage recognition methods based on deep learning.

Flah et al. [20]	1D-CNN	30	All nodes	86%
Azimi et al. [21]	Compressed data + 1D-CNN	30	All nodes	91.9%
Truong et al. [22]	1D-CNN + GRU	1	One joint	91.31%
Contrast model	GCN	30	All nodes	93.03%
Contrast model	LSTM	12	All nodes	93.37%
Proposed model	S_DFNN (GCN)	30	All nodes	99.57%

**Table 3:** The recognition accuracy of some models for frame structure node damage at different SNRs.

SNR (dB)	LSTM	GCN	S_DFNN (GCN)
80	0.93193	0.93095	0.99899
60	0.93151	0.93128	0.99899
40	0.93036	0.92960	0.99899
20	0.67137	0.82191	0.97757
15	0.31664	0.56939	0.80267
10	0.10607	0.24530	0.38684
5	0.04881	0.09224	0.14037

**Table 4:** Damage conditions of main girder units.

Damage Condition	Description of Damage Status
Condition 0 (non-destructive)	Structural baseline condition
Working condition 1	Replacement of unit D0 of the main beam with a type A damage unit
Condition 2	Replacement of unit D10 of the main beam with a type B damage unit
Condition 3	Replacement of unit D0 of the main beam with a type A damage unit Replacement of unit D10 of the main beam with a type B damage unit

**Table 5:** Parameter configuration of GCN model for damage identification of cable-stayed bridge model.

Parameter	Specific Values
First GCN layer	500
Second GCN layer	500
Third GCN layer	500
Dropout value	0.3
Training Optimizer	Adam

**Table 6:** Identification accuracy of structural damage of main beams by different models.

Model	Damage Unit Number				Total
	Non-Destructive	D0	D10	D0 + D10	
GCN	96.75%	88.75%	89.50%	79.25%	88.56%
S_DFNN (GCN)	99.99%	99.91%	99.97%	99.99%	99.97%

**Table 7:** Damage conditions of diagonal cable.

Damage Lasso Number	Description of Damage Status
non-destructive	structural baseline condition
LB1_W	No. LB1_W Slope cable slack 3 mm
LB9_W	No. LB9_W Slope cable slack 3 mm
LB1_W + LB9_W	No. LB1_W Slope cable slack 3 mm No. LB9_W Slope cable slack 3 mm

**Table 8:** Accuracy of different models in recognizing the damage of diagonal cable.

Mold	Damage Lasso Number				(Grand) Total
	Non-Destructive	LB1_W	LB9_W	LB1_W + LB9_W	
GCN	85.25%	77.75%	83.75%	64.75%	77.88
S_DFNN (GCN)	99.98%	99.99%	99.77%	98.75%	99.62%

## Data Availability

Data are contained within the article.
